# Leloir Glycosyltransferases and Natural Product Glycosylation: Biocatalytic Synthesis of the *C*-Glucoside Nothofagin, a Major Antioxidant of Redbush Herbal Tea

**DOI:** 10.1002/adsc.201300251

**Published:** 2013-08-20

**Authors:** Linda Bungaruang, Alexander Gutmann, Bernd Nidetzky

**Affiliations:** a Institute of Biotechnology and Biochemical Engineering,Graz University of Technology, Petersgasse 12, 8010 Graz, Austria, Fax: (+43)-316-873-8434; phone:(+43)-316-873-8400

**Keywords:** carbohydrates, *C*-glycosides, glycosyltransferases, natural products, UDP-glucose recycling

## Abstract

Nothofagin is a major antioxidant of redbush herbal tea and represents a class of bioactive flavonoid-like *C*-glycosidic natural products. We developed an efficient enzymatic synthesis of nothofagin based on a one-pot coupled glycosyltransferase-catalyzed transformation that involves perfectly selective 3′-*C*-β-d-glucosylation of naturally abundant phloretin and applies sucrose as expedient glucosyl donor. *C*-Glucosyltransferase from *Oryza sativa* (rice) was used for phloretin *C*-glucosylation from uridine 5′-diphosphate (UDP)-glucose, which was supplied continuously *in situ* through conversion of sucrose and UDP catalyzed by sucrose synthase from *Glycine max* (soybean). In an evaluation of thermodynamic, kinetic, and stability parameters of the coupled enzymatic reactions, poor water solubility of the phloretin acceptor substrate was revealed as a major bottleneck of conversion efficiency. Using periodic feed of phloretin controlled by reaction progress, nothofagin concentrations (45 mM; 20 g l^−1^) were obtained that vastly exceed the phloretin solubility limit (5–10 mM). The intermediate UDP-glucose was produced from catalytic amounts of UDP (1.0 mM) and was thus recycled 45 times in the process. Benchmarked against comparable glycosyltransferase-catalyzed transformations (e.g., on quercetin), the synthesis of nothofagin has achieved intensification in glycosidic product formation by up to three orders of magnitude (μM→mM range). It thus makes a strong case for the application of Leloir glycosyltransferases in biocatalytic syntheses of glycosylated natural products as fine chemicals.

Many bioactive natural products contain sugar molecule(s) as part of their structure.^1^ Their physiological activity, selectivity and pharmacological properties are often derived from the sugar component(s).^2^ Therefore, glycosylation is often central to a natural product’s efficacy in the particular application considered. Aside from therapeutic uses,^3^ glycosylated natural products have raised interest as functional food additives and cosmetic ingredients.^4^ Glycosylation pattern engineering is regarded as a highly promising way of functional diversification of natural products.2b,^3^ This might contribute to the creation of new bioactive substances and drug leads.

In nature, the selective modification of target compounds with sugars is catalyzed by glycosyltransferases (EC 2.4).^5^ These enzymes use an activated donor substrate, typically a nucleoside diphosphate (NDP)-sugar, for transfer of the glycosyl residue onto a specific position of an acceptor molecule. Glycosyltransferases display splendid regioselectivity and stereochemical control in the transformations catalyzed,^6^ and they are therefore widely recognized as highly valuable glycosylation catalysts.^7^ However, synthetic applications of glycosyltransferases have so far been quite restricted due to complexities of the enzymes (e.g., low specific enzyme activity and stability)^8^ and the supply of donor and acceptor substrates for the enzymatic reactions.^9^

The majority of natural product glycosylations involve *O*-glycosidic bonds. Glycosylations at carbon, by contrast, are relatively rare and to date only a small number of natural *C*-glycosyltransferases have been reported.^10^ The *C*-glycosidic linkage displays outstanding resistance to chemical or enzyme-catalyzed hydrolysis, surpassing that of the corresponding *O*-glycosidic linkage by a large amount.^11^ *C*-glycosides have therefore attracted considerable attention for functional substitution of physiologically active *O*-glycosidic compounds having low *in vivo* lifetimes.^12^

Aryl glucosides derived from flavonoid-like aglycones (Scheme  1) present a very interesting class of plant natural products that may involve either a *C*- or an *O*-glycosidic linkage.^13^ They show a highly significant profile of biological activities that typically include strong antioxidant and radical scavenger functions, but also comprise antiviral and cytotoxic effects.^14^ Because product isolation directly from the plant is often impractical, compounds must also be prepared by bottom-up synthesis. Despite notable recent advancements,^15^ chemical methodologies involve multiple steps and are therefore generally neither atom-efficient nor high-yielding. We demonstrate in this study that single-step glycosyltransferase-catalyzed transformation *in vitro* presents a powerful tool for aryl *C*-glycoside synthesis. We show that when offered the dihydrochalcone phloretin as acceptor, *C*-glycosyltransferase from rice (*Oryza sativa*; *Os*CGT) reacts with uridine 5′-diphosphate (UDP)-glucose to give the 3′-*C*-aryl β-d-glucoside nothofagin (Scheme  1) as a single transfer product. Nothofagin is a natural substance found in redbush herbal tea and represents a structural class of bioactive aryl *C*-glycosides.^16^ For efficient nothofagin synthesis, we coupled the *C*-glucosyltransferase reaction to enzymatic *in situ* supply of the glucosyl donor substrate (Scheme  1): UDP-glucose is produced from sucrose and UDP using recombinant sucrose synthase from soybean (*Glycine max*; *Gm*SuSy). While the applied internal UDP-glucose regeneration is known in principle and has been applied to enzymatic reactions involving different glycosyl donor substrates,^17^ a critical test of its performance capability in the synthesis of natural product glucosides such as nothofagin17e–h remains outstanding. We show the application of comprehensive step-by-step reaction engineering to overcome complexities inherent to this and similarly coupled glycosyltransferase systems and report an efficient, high-yielding biocatalytic production of nothofagin.

**Sceheme 1:**
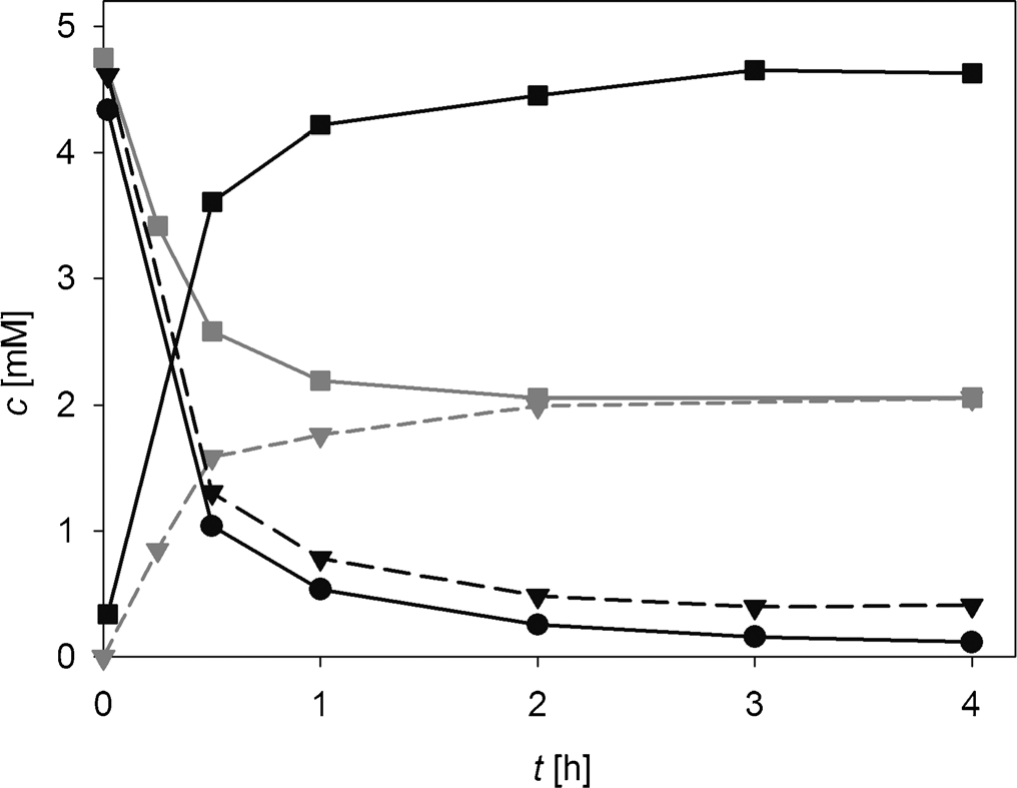
Synthesis of nothofagin is achieved by enzymatic *C*-glucosylation of phloretin from sucrose *via* UDP-glucose. *Os*CGT and *Gm*SuSy are telescoped in one pot, and the reaction proceeds in the presence of catalytic amounts of UDP. Fructose is the only by-product formed. The overall equilibrium lies far on the *C*-glycoside product side, driven by the reaction of *Os*CGT and the presence of sucrose in excess.

We first examined reactions of *Os*CGT and *Gm*SuSy separately and determined their kinetic and thermodynamic characteristics. Enzymes were obtained from *Escherichia coli* expression cultures and purified to apparent homogeneity by Strep-tag affinity chromatography, as described in the Supporting Information (Methods, Figure S1). Their activities were determined using enzymatic or HPLC-based assays (Supporting Information, Methods). The reaction of *Os*CGT was monitored with an HPLC assay capable of distinguishing between nothofagin and potential alternative products resulting from *O*-glucosyl transfer at the 2′ or 4′ position of the acceptor (Supporting Information, Figure S2).^18^ *Os*CGT displayed absolute selectivity (within an error limit of ≤0.5%) for 3′-*C*-glycosylation of phloretin. We determined pH-activity dependencies for *Gm*SuSy (sucrose cleavage and synthesis) and *Os*CGT (nothofagin synthesis) at 30 °C. The resulting pH profiles revealed suitable overlap of the enzyme activities in the pH range 6.5–8.0 (Supporting Information, Figure S3). The results shown in Figure 1 indicate that *C*-glucosylation of phloretin at pH 7.5 resulted in high conversion of substrates (≥95 %). Moreover, the enzymatic reverse reaction with nothofagin and UDP was not detectable under these conditions (Supporting Information, Figure S4). We concluded, therefore, that synthesis of nothofagin by *Os*CGT proceeds without critical thermodynamic limitations. The equilibrium for sucrose conversion is pH-dependent, and a low pH of 6 or smaller is known to favor the formation of UDP-glucose.^19^ Figure 1 shows that at pH 7.5, the equilibrium constant (*K*_eq_) for conversion of sucrose and UDP had a value of 0.49. However, thermodynamic constraints on the supply of UDP-glucose at elevated pH can be eliminated effectively using sucrose in excess. We therefore performed our conversion studies at pH 7.5 and 30 °C where both glycosyltransferases showed useful activity and stability (Table 1) and quantitative transformation of sucrose into nothofagin was feasible.

**Figure 1:**
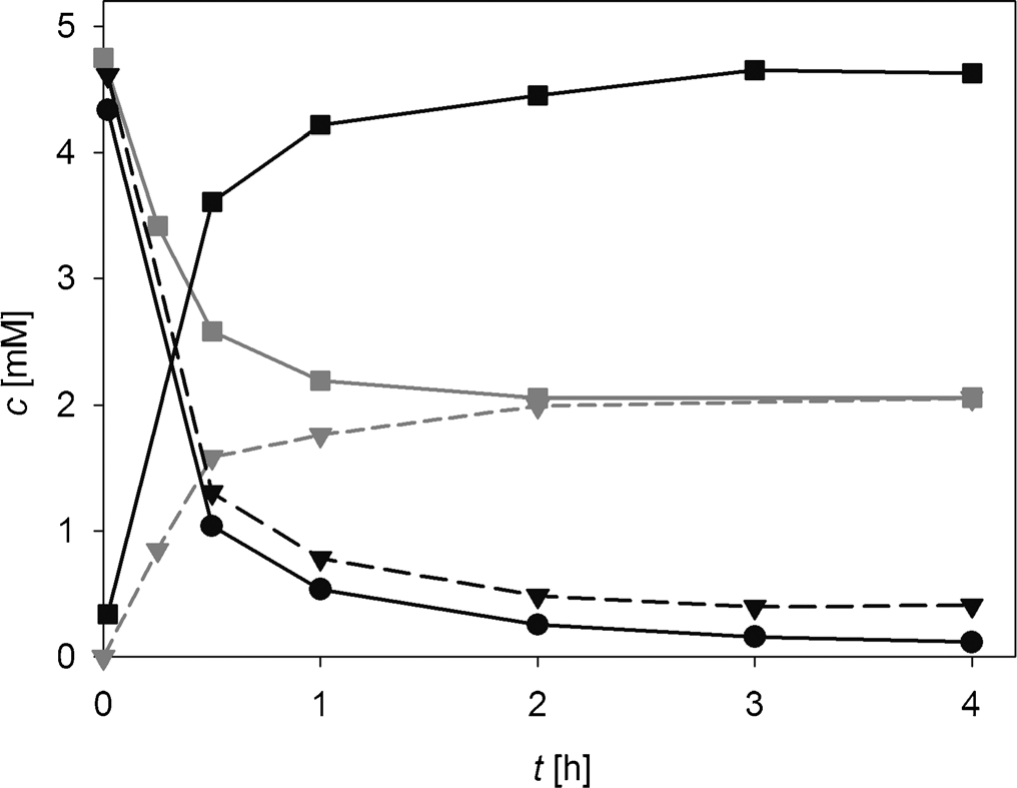
Time course analysis for individual enzymatic reactions catalyzed by *Os*CGT and *Gm*SuSy at pH 7.5 and 30 °C. Nothofagin synthesis by *Os*CGT (black symbols): 80 mU mL^−1^, 5 mM phloretin (triangle, dashed line), 4.75 mM UDP-glucose (circle, solid line), nothofagin (square, solid line). Reactions of *Gm*SuSy (grey symbols): 50 mU mL^−1^, 5 mM of each substrate, UDP-glucose in sucrose synthesis (squares, solid line) and cleavage (triangles, dashed line).

**Table 1:** Characterization of glycosyltransferases and their reactions.[a]

Parameter	*Gm*SuSy		*Os*CGT
	Synthesis	Cleavage	
*K*_M sucrose_ [mM]	–	25.5±3.3[b]	–
*K*_M UDP_ [mM]	–	0.13±0.02[b]	–
*K*_M fructose_ [mM]	3.0±0.4[b]	–	
*K*_M UDP−glucose_ [mM]	0.14±0.03[b]	–	0.024±0.004
*K*_M phloretin_ [mM]	–	–	0.009±0.003
*k*_cat_ [s^−1^]	7.5±0.4	9.3±0.3	4.4±0.3
Spec. act. [U mg^−1^]	4.8±0.2	5.9±0.2	5.1±0.3
*K*_eq_		0.49±0.01^[c,e]^	>400^[d,e]^
*t*_1/2_ [h]		18.8±0.9[f]	13.8±1.2[g]

[a] 30 °C, 50 mM HEPES pH 7.5, 20% (v/v) DMSO.

[b] 30 °C, 20 mM HEPES, pH 7.5.

[c] Cleavage direction (conversion of sucrose and UDP).

[d] Glycosylation direction (conversion of phloretin and UDP-glucose).

[e] Calculated from data in Figure 1.

[f] 30 °C, 50 mM HEPES pH 7.5, 20% (v/v) DMSO, 100 mM sucrose.

[g] 30 °C, 50 mM HEPES pH 7.5, 20% (v/v) DMSO, 5 mM phloretin.

The low water solubility of non-carbohydrate acceptor substrates is an important issue for carrying out natural product glycosylations *in vitro*. In the case of the barely water-soluble phloretin, use of an organic cosolvent was essential to enhance the acceptor substrate availability in *C*-glycosylations catalyzed by *Os*CGT. Whereas both ethanol and DMSO up to 20% by volume caused only minor interference with *Os*CGT activity, *Gm*SuSy displayed a low cosolvent tolerance and its activity was almost completely (≥85%) lost in the presence of 15% ethanol. DMSO was less strongly affecting the activity of *Gm*SuSy and around 65% of the specific enzyme activity in purely aqueous buffer were retained in 20% DMSO. Furthermore, *Os*CGT stability and phloretin solubility were superior in DMSO as compared to ethanol. All conversion experiments were therefore performed in 20% DMSO, and the maximum concentration of dissolved phloretin was around 10 mM under these conditions.

Kinetic characterization of *Gm*SuSy (sucrose conversion) and *Os*CGT (nothofagin synthesis) was done at pH 7.5 and results are summarized in Table 1 along with the relevant enzyme stability and reaction thermodynamic parameters under these conditions. Both enzymes showed useful specific activities (≥5 units mg^−1^ protein) and were sufficiently stable under the reaction conditions with half-lives of around 19 (*Gm*SuSy) and 14 h (*Os*CGT). The Michaelis–Menten constant (*K*_M_) of *Gm*SuSy for sucrose exceeds the corresponding *K*_M_ for UDP by two orders of magnitude. *K*_M_ values of *Os*CGT are also much lower than the sucrose *K*_M_. Therefore, this implies that relatively high sucrose concentrations should be used in the coupled reaction to partly saturate and thus make optimum use of the *Gm*SuSy activity present. The kinetic requirements of SuSy are therefore in good accordance with the notion of using an excess of sucrose to drive the overall conversion. We noticed that the *K*_M_ for UDP was 26-fold higher in our recombinant preparation of *Gm*SuSy as compared to the enzyme isolated from the native source.^20^ This large difference in apparent UDP binding affinity might be due to effects of post-translational modification (e.g., covalent phosphorylation) that have been described for sucrose synthases in plants^21^ and that may not occur in *E. coli*. The *K*_M_ for UDP-glucose was also strongly elevated (12-fold) in recombinant as compared to native *Gm*SuSy while, interestingly, the *K*_M_ values for sucrose and fructose were not affected in the recombinant enzyme. Differences between recombinant and native *Gm*SuSy were not further pursued in this study.

We performed synthesis experiments in which the initial concentration of sucrose (5–500 mM; 0.5 mM UDP) or UDP (0.005–1 mM; 100 mM sucrose) was varied while enzymatic activity (10 mU mL^−1^ *Os*CGT/*Gm*SuSy) and phloretin concentration (5 mM) were constant. The nothofagin production rate (*r*_P_) was measured, and results are depicted in Figure 2. Dependence of *r*_P_ on the sucrose concentration was hyperbolic, with a half-saturation constant (27 mM) comparable to the *K*_M_ of *Gm*SuSy for sucrose. Therefore, it appears to primarily reflect saturation behavior of *Gm*SuSy, as noted above (Table 1). The dependence of *r*_P_ on the UDP concentration was likewise hyperbolic, with a half-saturation level (51 μM) in between the *K*_M_ values for UDP and UDP-glucose. Figure 2B) indicates that the applied “nothofagin synthase” activity, derived from the combined activities of *Os*CGT and *Gm*SuSy, was therefore utilized best at a UDP concentration of 0.5 mM or higher. Using a phloretin concentration of 5 mM, this limited the maximum number of UDP-glucose regeneration cycles (*RC*_max_) to 10 (=5/0.5). It would certainly be possible to further increase this *RC*_max_ value by decreasing the UDP concentration relative to the phloretin concentration, but this would probably have to occur at the expense of a significant loss in *r*_P_. It is interesting that at the lowest UDP concentration used in Figure 2 (5 μM), the observed *r*_P_ was still 10% of its maximum value at saturation with UDP.

**Figure 2:**
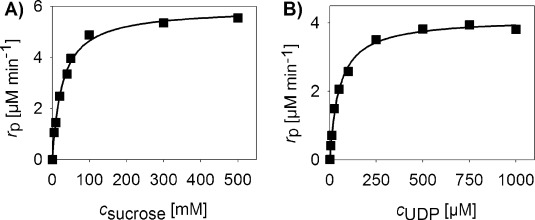
The nothofagin production rate (*r*_p_) in a coupled-enzyme reaction (10 mU mL^−1^ *Os*CGT/*Gm*SuSy, 5 mM phloretin) depends on variation of A) the sucrose concentration (0.5 mM UDP) and B) the UDP (100 mM sucrose) concentration. Note: because sucrose was not fully saturating in B) the achieved *r*_p_ at high UDP is slightly lower than in A).

Aside from cost-efficient supply of the UDP-glucose donor substrate and favorable thermodynamic effects resulting from the use of high sucrose concentrations, a glucosyltransferase reaction might benefit from its coupling to the SuSy reaction kinetically. Pronounced end-product inhibition by micromolar concentrations of UDP is quite common among flavonoid *O*-glucosyltransferases17e,g,h and imposes severe restrictions on the direct synthetic use of these enzymes which could be decreased by continuous removal of the UDP released.17e,^22^ We tested the influence of UDP inhibition on nothofagin production by comparing *Os*CGT (50 mU mL^−1^) conversion of 5 mM phloretin (6 mM UDP-glucose, 100 mM sucrose) in absence and presence of *Gm*SuSy (50 mU mL^−1^) (Figure 3A). Although in both reactions quantitative conversion (>99.5%) was achieved *r*_P_ showed a stronger decrease above ∼75% conversion (2 h) without *Gm*SuSy and final conversions (>99%) were only reached after more than 10 h compared to less than 6 h in the presence of *Gm*SuSy. This corresponds to an approximately two-fold gain in space-time yield in nothofagin production (mM product formed/time consumed) resulting from the coupling of *Os*CGT and *Gm*SuSy reactions. UDP-glucose depletion could be excluded as an explanation for the reduction of *r*_P_ in the absence of *Gm*SuSy due to excess of UDP-glucose (1 mM, 40-fold *K*_M_) which was monitored throughout the conversion. Although end product inhibition at low millimolar UDP concentrations was less critical for *Os*CGT than for *O*-glycosyltransferases, *in situ* removal of UDP remains an essential feature for general application of SuSy as UDP-glucose recycling system for high level glycoside production.

**Figure 3:**
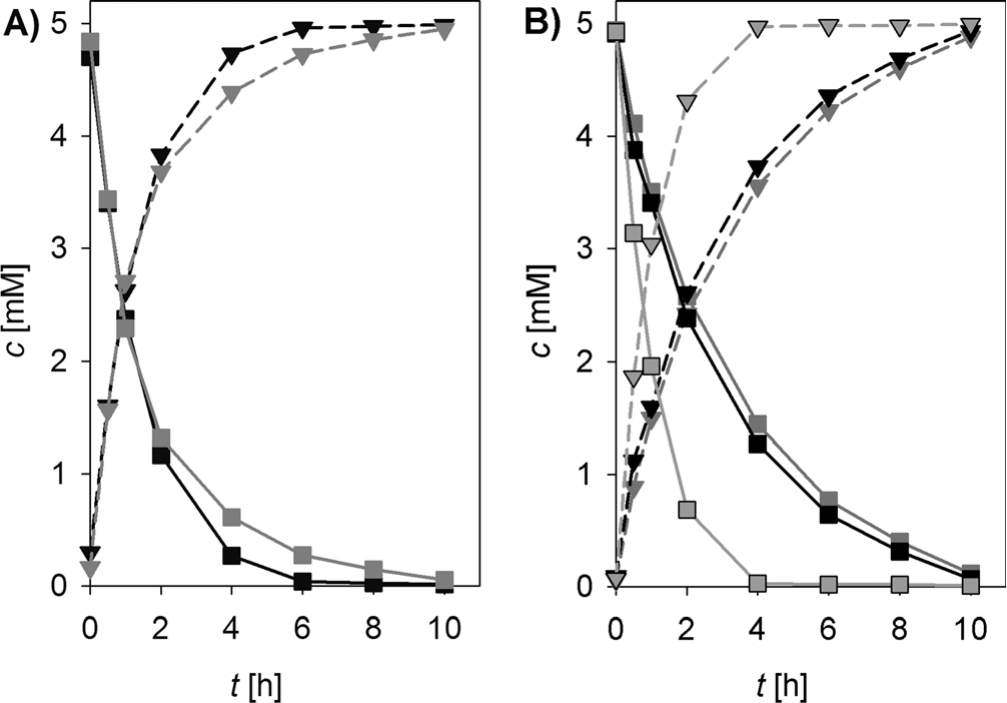
Conversions of 5 mM phloretin (square) to nothofagin (triangle) by *Os*CGT (100 mM sucrose): A) Using 50 mU ml^−1^ *Os*CGT and 6 mM UDP-glucose in the absence (grey) and presence (black) of 50 mU mL^−1^ *Gm*SuSy; B) variation of *Os*CGT and *Gm*SuSy activity in coupled conversions (0.5 mM UDP): 50 mU mL^−1^ *Os*CGT/*Gm*SuSy (dark grey); 50 mU mL^−1^ *Os*CGT and 250 mU mL^−1^ *Gm*SuSy (black); 250 mU mL^−1^ *Os*CGT and 50 mU mL^−1^ *Gm*SuSy (light grey, black edge).

Conversion rates were slightly lower when 5 mM UDP-glucose were replaced with 0.5 mM UDP in a coupled glycosyltransferase conversion (50 mU mL^−1^ *Os*CGT/*Gm*SuSy) (Figure 3B; Supporting Information, Figure S5) and complete conversion was only reached after 10 h. Also a 5-fold excess of *Gm*SuSy (250 mU mL^−1^) over *Os*CGT (50 mU mL^−1^) did not improve nothofagin production significantly. On the other hand a five-fold excess of *Os*CGT (250 mU mL^−1^) over *Gm*SuSy (50 mU mL^−1^) drastically increased the conversion resulting in complete conversion after only 4 h. Thereby *C*-glucosylation was identified as a rate-limiting step at the applied conditions. UDP-glucose levels of roughly 0.1 mM throughout all three conversions coincide with the finding that the UDP-glucose supply was not critical in the coupled conversions. Furthermore, it is worth remarking that the produced nothofagin was stable in all conversions, suggesting that the *C*-glucoside synthesis is conveniently performed under thermodynamic control and in the apparent absence of chemical or enzyme-catalyzed side reactions.

Considering that solubility of phloretin was markedly enhanced (≥5-fold) upon its *C*-glycosylation, we raised the initial concentration of phloretin in various steps to 30 mM, thereby exceeding the solubility limit of the acceptor substrate by at least 3-fold. We figured that insoluble phloretin might still be useful for the continuous *in situ* supply of acceptor substrate when gradual transformation of the dissolved phloretin occurred in the enzymatic reaction (100 mM sucrose, 0.5 mM UDP, 190 mU mL^−1^ *Os*CGT, 120 mU mL^−1^ *Gm*SuSy). Figure 4 shows that nothofagin production could not be upheld under conditions of insoluble acceptor being present (≥10 mM) and also the final product concentration after 24 h was strongly decreased in clear dependence on the phloretin concentration. Using 10 mM phloretin, which was initially dissolved completely, we noticed precipitation of the acceptor substrate over time, limiting the maximum amount of nothofagin obtainable in the reaction under these conditions despite complete conversion of all soluble phloretin (Figure 4B). Furthermore, initial rate studies of *Os*CGT revealed substrate inhibition (*K*_i_∼5 mM) which clearly effected conversion at phloretin concentrations above 1 mM.

**Figure 4:**
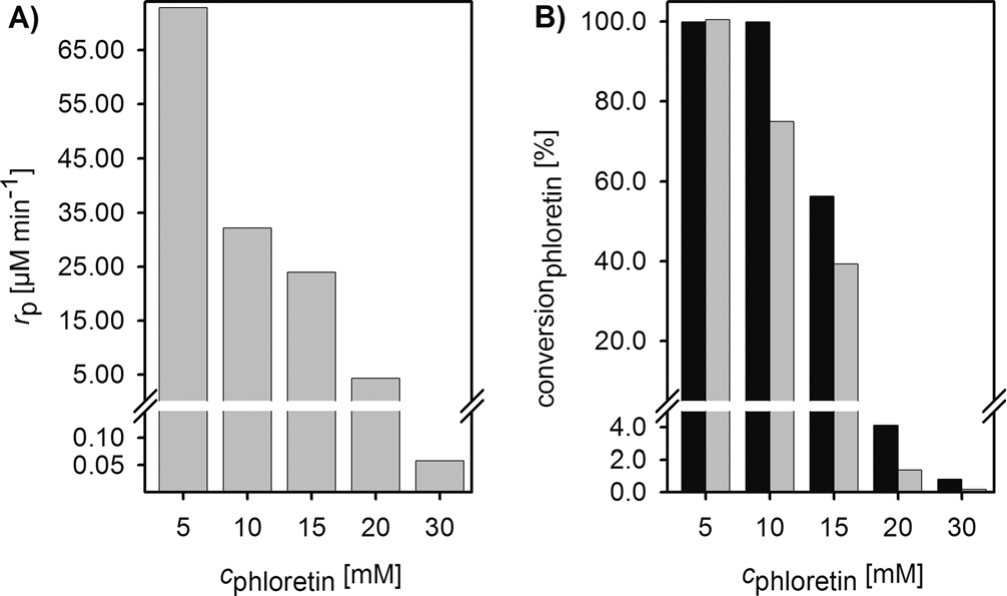
Batch conversions at different phloretin concentrations (100 mM sucrose, 0.5 mM UDP, 190 mU mL^−1^ *Os*CGT, 120 mU mL^−1^ *Gm*SuSy); A) Nothofagin production rate decreases at elevated phloretin concentrations; B) final conversion of soluble (black) and total applied (grey) phloretin after 24 h is limited at high phloretin concentrations by substrate inhibition and solubility.

To nevertheless increase the end concentration of nothofagin in the enzymatic reaction, we changed the operation mode from batch to fed-batch, adding fresh phloretin to a concentration of 5 or 10 mM once the acceptor substrate had been depleted. Up to 8 rounds of phloretin addition were made, using a highly concentrated stock solution of 500 mM phloretin in pure DMSO to minimize the resulting volume change. Table 2 presents a summary of conditions and results and Figure 5 shows a reaction time course where in each round fresh enzyme was supplied together with 5 mM acceptor to the reaction. Feeding the phloretin acceptor was generally quite effective in enhancing nothofagin production. However, the phloretin conversion rate decreased strongly in dependence on the total amount of acceptor added to the reaction, so that without enzyme feed, the maximum concentration of nothofagin was just around 20 mM (Table 2). Co-addition of enzyme alleviated restrictions on the product concentration (substrate conversion), which probably resulted from the combined effect of true enzyme activity loss and product inhibition. *Gm*SuSy is inhibited by d-fructose with a reported *K*_i_ of 9 mM.^20^ This could also explain why lower total turnover numbers (ttn) were obtained with enzyme feed (∼10,000) than without (∼20,000). We also noticed the requirement to carefully control the phloretin feed to keep the acceptor concentration well below its solubility limit during the reaction. Figure S6 (Supporting Information) depicts *in situ* precipitation of phloretin under conditions where the acceptor feeding rate was not matched to the enzymatic consumption rate. Phloretin precipitation was clearly reflected in the mass balance in solution (Table 2).

**Table 2:** Nothofagin synthesis using controlled feed of phloretin and enzyme.[a]

*Δc*_phloretin_[b] [mM]	5	5	10	10
Enzyme feed	no	yes	no	yes
*c*_phloretin_[c] [mM]	20	45	40	60
Vol. act[c] [mU mL^−1^]	100	550	100	600
*t* [h]	27	135	42	120
*c*_nothofagin_[d] [mM]	14.6	44.1	19.7	46.6
Conversion[e] [%]	88	98	63	90
Precipitation [mM][f]	3.5	<0.1	8.9	8.1
ttn[g] (*Gm*SuSy/*Os*CGT) [1⋅10^3^]	18/16	10/9	25/21	10/8

[a] 300 mM sucrose, 1 mM UDP, 30 °C, 50 mM HEPES pH 7.5, 20% DMSO.

[b] Amount of phloretin added per feeding.

[c] Total amount of phloretin/enzyme activity added.

[d] Final nothofagin concentration in solution.

[e] Based on final nothofagin and phloretin concentrations in solution.

[f] Difference total fed phloretin and final soluble nothofagin and phloretin.

[g] Total turnover number (mM nothofagin/mM total enzyme added).

**Figure 5:**
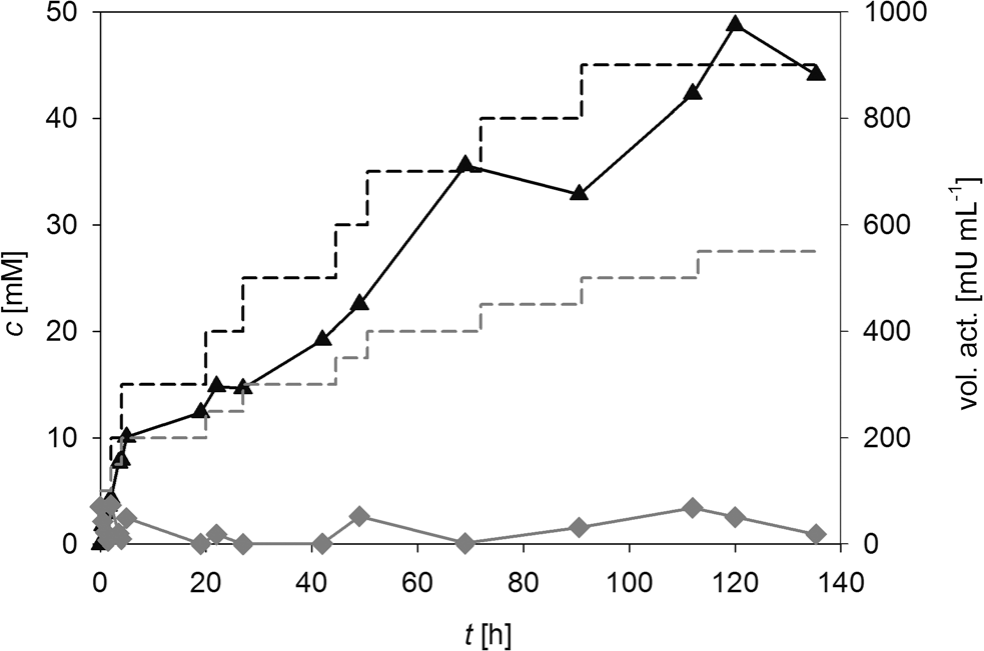
Controlled feeding of phloretin is useful to enhance the nothofagin concentration in the biocatalytic synthesis. *Reaction conditions:* 100 mU mL^−1^ *Os*CGT/*Gm*SuSy, 5 mM phloretin, 300 mM sucrose, 1 mM UDP. After acceptor substrate depletion, 5 mM phloretin and 50 mU mL^−1^ *Os*CGT/*Gm*SuSy were added. Symbols: phloretin added (black dashed), *Os*CGT/*Gm*SuSy added (grey dashed), nothofagin (black), phloretin (grey)

Applying a suitable stepwise feeding of phloretin (5 or 10 mM) that included supplementation with fresh enzyme, it was possible to accumulate nothofagin in a concentration of around 45 mM, equivalent to 20 g l^−1^ (Figure 5, Table 2) which corresponded to *RC*_max_ values of approximately 45. It has to be noted that only with addition of enzyme and a low acceptor feed (5 mm) (Figure 5) could precipitation be avoided and almost quantitative conversion (∼98%) was achieved. Nothofagin was isolated from reaction mixtures in a single step using preparative reversed phase C-18 HPLC with water to methanol gradient elution. Typically more than 80% of the initially applied phloretin (≥25 mg) could be recovered as highly pure nothofagin (Supporting information, Figure S8C). The isolation procedure is simple and not limited in scale.

Glycosyltransferases currently underachieve to a large extent their often-quoted high potential as synthetically usable glycosylation catalysts.^9^ Notable exceptions in the field of complex oligosaccharides notwithstanding,17a,d,^22^ glycosyltransferase transformations have been realized almost exclusively at the analytical or minute preparative scale.^23^ Glycosylations of similar poorly water-soluble natural product core structures such as the flavonoid quercetin have furnished hardly more than micromolar concentrations of the desired glycosidic compound.17g,h,^24^ On comparison with the literature, therefore, the herein described enzymatic process of glucosylation of phloretin from sucrose stands out due to intensification, by up to three orders of magnitude, in terms of the product concentration that it has achieved for a coupled glycosyltransferase-catalyzed conversion. Furthermore, with the notable exception of the recently reported application of *Os*CGT for the production of nothofagin (∼200 μM) and 2-hydroxynaringenin *C*-glucoside using engineered *S. cerevisiae* strains for whole cell conversions23c this is the first synthetic use of a *C*-glucosyltransferase. The here reported nothofagin process features efficient assembly of isolated *C*-glucosyltransferase in one pot with an adaptable module for UDP-glucose supply from sucrose, which serves as a highly expedient glucosyl donor for the overall conversion. We show that systematic analysis of thermodynamic conditions, kinetic properties of the glycosyltransferases and their stabilities is the key for identifying and thus eliminating critical constraints on the multi-component reaction system. Integration of biochemical optimization with reaction engineering was essential to overcome the restriction of acceptor substrate solubility. The number of UDP-glucose regeneration cycles was brought into a range (around 50) where one begins to truly capitalize on the coupling with the SuSy reaction. Reported *RC*_max_ values in literature are by far too small^[17e 17g,17h]^ to justify enzymatic recycling of UDP-glucose. However, costs of the donor substrate are expected to prohibit the direct use of UDP-glucose for synthesis. Considering that unprocessed redbush tea contains nothofagin to just about 4.31 g kg^−1^ freeze-dried matter,^25^ the high-yielding enzymatic synthesis developed herein is expected to remove compound availability as a critical bottleneck of the various medical and food-related applications of this aryl-*C*-glucoside.14b,^16^,^26^ This study therefore makes a strong and so far missing case for the application of glycosyltransferases in biocatalytic synthesis of glycosylated natural products as fine chemicals.

## Experimental Section

### Coupled Enzymatic Conversions

Unless otherwise mentioned, standard reaction mixtures contained 5 mM phloretin, 100 mM sucrose, 0.5 mM UDP, 13 mM MgCl_2_, 50 mM KCl, 0.13% (w/v) BSA, and 20% (v/v) DMSO in 50 mM HEPES buffer pH 7.5. Reactions were started by the addition of the indicated amounts of *Os*CGT and *Gm*SuSy. Enzymatic reactions were performed in 1.5 mL Eppendorf tubes at 30 °C using a thermomixer comfort for temperature control and agitation at 400 rpm. Samples were mixed with an equal volume of acetonitrile to stop the reaction. Precipitated protein was removed by centrifugation (13,200 rpm). The supernatant was analyzed using a reversed phase C-18 HPLC-assay.^18^ All compounds of the reaction were analyzed. Reported conversions are confirmed by closed mass balance.

A detailed description of all experimental procedures can be found in the Supporting Information, which comprises: cloning of *Gm*SuSy; expression and purification of *Os*CGT and *Gm*SuSy; enzyme assays; details of the biocatalytic transformations performed; product isolation and identification; and analytical methods used. Any associated references are also given.

## Notes

As a service to our authors and readers, this journal provides supporting information supplied by the authors. Such materials are peer reviewed and may be re-organized for online delivery, but are not copy-edited or typeset. Technical support issues arising from supporting information (other than missing files) should be addressed to the authors.
