# Supplementary material for: Leloir Glycosyltransferases and Natural Product Glycosylation: Biocatalytic Synthesis of the C-Glucoside Nothofagin, a Major Antioxidant of Redbush Herbal Tea
Source: Adv Synth Catal. 2013 Aug 20;355(14-15):2757–63. doi: 10.1002/adsc.201300251 (PMC3883091; doi:10.1002/adsc.201300251)
Supplement: Supplementary file 1 [file adsc0355-2757-sd1.pdf]

Advanced Synthesis & Catalysis

Supporting Information

© Copyright Wiley-VCH Verlag GmbH & Co. KGaA, 69451 Weinheim, 2013

SUPPORTING INFORMATION

Leloir glycosyltransferases and natural product glycosylation: biocatalytic synthesis of the *C*-glucoside nothofagin, a major antioxidant of redbush herbal tea

Linda Bungaruang,[+a] Alexander Gutmann,[+a] and Bernd Nidetzky[a,*]

[a] Institute of Biotechnology and Biochemical Engineering, Graz University of Technology, Petersgasse 12, 8010 Graz, Austria
Fax: (+43)-316-873-8434; phone:(+43)-316-873-8400; e-mail: bernd.nidetzky@tugraz.at

[+] These authors contributed equally.

Table of contents:

1 Methods **S2**

1.1 Chemicals and reagents **S2**

1.2 Preparation of purified *Os*CGT and *Gm*SuSy **S2**

1.3 Determination of enzymatic activities and conversions **S3**

1.4 Characterization of single enzymes **S5**

1.5 *Os*CGT conversions coupled with *Gm*SuSy **S7**

1.6 Product isolation and identification by HPLC and NMR **S7**

2 Results **S8**

3 References **S13**

1 Methods

1.1 Chemicals and reagents

Unless otherwise indicated, all chemicals were purchased from Sigma-Aldrich in the highest purity available. Phloretin (98% purity) was obtained from AK Scientific. Enzymes for DNA manipulation and GeneJET™ Plasmid Miniprep Kit were from Fermentas. *Strep*-Tactin® Sepharose® and desthiobiotin were from IBA. BCA assay kit was purchased from Thermo Scientific.

1.2 Preparation of purified *Os*CGT and *Gm*SuSy

Construction of expression strains

The *Os*CGT gene (GenBank: FM179712) was received as a kind gift from the group of Prof. Robert Edwards (Centre for Bioactive Chemistry, Durham University, UK). It was provided in a pET-STRP3 vector which is a custom made derivative of pET-24d that enables protein expression with N-terminally fused *Strep*-tag II.[1, 2] The codon optimized gene of *Gm*SuSy (GenBank: AF030231) was synthesized with flanking *Nde*I and *Xho*I restriction sites and cloned in the plasmid pUC57 by GenScript. The gene was cut out using *Nde*I and *Xho*I restriction enzymes and inserted into the respective sites of the pET-STRP3 vector for expression as fusion protein with N-terminal *Strep*-Tag II.
Expression strains were created by transformation of electro-competent *E. coli* BL21-Gold (DE3) cells. The correct sequences were verified by sequencing the complete genes.

Protein expression and purification

The described *E. coli* strains were cultivated in 1 L baffled shaking flasks containing 250 mL Luria Bertani (LB) medium with 50 µg mL^{-1} kanamycin on a rotary shaker at 37℃ and 120 rpm. Protein expression was induced at an optical density at 600 nm between 0.8 and 1.0 by addition of 0.2 mM isopropyl β-D-1-thiogalactopyranoside (IPTG) and the expression was carried out overnight at 25 ℃. The cells were harvested by 30 min centrifugation at 4℃ and 5000 rpm. After resuspension in water they were stored at -70℃ until disruption by repeated passage through a cooled French press at 100 bar. Cell debris was removed by centrifugation for 45 min at 4℃ and 13200 rpm.
The *Strep*-tag II fusion proteins were purified from the cell extract by affinity chromatography on *Strep*-Tactin® Sepharose® columns according to instructions of the manufacturer IBA. The columns had a volume of 3 mL and were operated by gravity flow. Equilibration of the column was done with 3 column volumes (CVs) of washing buffer W (100 mM Tris/HCl pH 8, 150 mM NaCl, 1 mM EDTA). The cell extract was diluted twofold with buffer W and filtrated through a 1.2 µm cellulose-acetate syringe filter before loading on the column. After washing with 5 CVs of buffer W the proteins were eluted with 3 CVs buffer E (100 mM Tris/HCl pH 8, 150 mM NaCl, 1 mM EDTA, 2.5 mM desthiobiotin) whereas the first 0.5 CVs were discarded and the rest was pooled. The column was regenerated using 15 CVs of buffer R (100 mM Tris/HCl pH 8, 150 mM NaCl, 1 mM EDTA, 1 mM hydroxy-azophenyl-benzoic acid) and equilibrated with 10 CVs of buffer W. Between the purification of different enzymes the columns were washed with 8 M guanidine-HCl to rule out cross-contaminations. Eluted enzymes were

concentrated and buffer exchanged to 50 mM HEPES buffer pH 7.5 using centrifugal concentrators with a Molecular Weight Cut Off of 10 kDa. Enzymes were aliquoted and small aliquots were thawed for experiments as required and did not undergo multiple freeze-thaw cycles.

Protein concentration and purity

Protein concentrations were determined using the BCA method with bovine serum albumin (BSA) as standard. Purities were estimated by sodium dodecyl sulfate polyacrylamide gel electrophoresis (SDS-PAGE) analyses and Coomassie Blue staining.

1.3 Determination of enzymatic activities and conversions

HPLC-based determination of phloretin and nothofagin (*Os*CGT activity)

A HPLC-assay was used for determination of dihydrochalcone concentrations (phloretin and nothofagin) and it was also applied as main assay for measurement of nothofagin production rates in *Os*CGT conversions. Reactions were performed in 1.5 mL reaction tubes at 30°C in a thermomixer at 400 rpm. Concentrations of UDP-glucose and phloretin as well as the exact buffer conditions are listed at the respective experiment. Reactions were started with the addition of *Os*CGT and stopped by mixing an aliquot of 100 µL with 100 µL acetonitrile. At least four samples (typically every 20 min) were taken during the linear initial rate to determine activities. Precipitated protein was removed by centrifugation for 20 min at room temperature and 13200 rpm. Depending on the concentrations 5 or 10 µL of the supernatant were used for analysis on an Agilent 1200 HPLC equipped with a Chromolith® Performance RP-18e endcapped column (100-4.6 mm) from Merck. The column was thermostatically controlled at 35°C and the separation was monitored by UV detection at 288 nm. Separation of phloretin and its glycosides was achieved by following method using water with 0.1 % TFA (trifluoroacetic acid) as solvent A and acetonitrile with 0.1% TFA as solvent B, respectively. A 7.5 min long linear gradient from 20 to 47.5% B (1 mL min^{-1}) was used for product separation. It was followed by 0.05 min of a linear gradient from 47.5 to 100 % B (1 mL min^{-1}) and 1.45 min of isocratic flow at 100% B (1.5 mL min^{-1}) to wash off hydrophobic compounds. After a 0.05 min linear gradient from 100 to 20 % B (1.5 mL min^{-1}) an isocratic flow of 2.45 min at 20% B (1.5 mL min^{-1}) was applied to equilibrate the column.
One Unit of *Os*CGT used in batch or fed-batch conversions for nothofagin production was defined as the amount of enzyme producing 1 µmol nothofagin per minute under following conditions: 0.6 mM UDP-glucose, 5 mM phloretin, 50 mM HEPES, pH 7.5, 13 mM $MgCl_2$, 50 mM KCl, 0.13% BSA and 20% (v/v) DMSO.

HPLC-based determination of UDP and UDP-glucose

Determination of UDP and UDP-glucose concentrations was done by HPLC using the sample preparation protocol with acetonitrile addition described for dihydrochalcone measurements. 10 µL sample were applied on an Agilent 1200 HPLC equipped with a Chromolith® Performance RP-18e endcapped column which was thermostatically controlled at 30°C. Separation was monitored by UV detection at 254 nm. Using 20 mM potassium phosphate buffer pH 6.8 with 2 mM tetrabutylammonium hydrogen sulfate as solvent A and acetonitrile as solvent B following gradient was applied with

constant flow rate of 2 mL min^{-1}: 3 min of a linear gradient from 0 to 2% B were followed by a 7 min long linear gradient from 2 to 25% B to separate UDP-glucose from UDP. During 2 min of isocratic flow at 25% B hydrophobic compounds were washed off and after a 1 min long gradient from 25 to 0%, 2 min of isocratic flow at 0% B were applied to equilibrate the column.

Photometric assay for *Os*CGT and *Gm*SuSy sucrose synthesis activity

Activity of *Os*CGT as well as *Gm*SuSy activity in sucrose synthesis direction were also determined with a photometric assay using a modified spectrophotometric method.[3] It is based on coupling of UDP formation through pyruvate kinase (PK) and lactic dehydrogenase (LD) to equimolar NADH (ε = 6220 M^{-1} cm^{-1}) depletion which can be followed photometrically at 340 nm. The assay was performed in a discontinuous way whereas the reaction conditions for *Os*CGT measurements were as described for the HPLC based assay. Concentrations of UDP-glucose and fructose for *Gm*SuSy activity measurements are listed together with the used buffer in the corresponding sections. Samples of 150 µL were stopped by heating to 95°C for 5 min. After removing precipitated protein by centrifugation at room temperature and 13200 rpm for 20 min, 100 µL of the supernatant were mixed in a Half Micro Cuvette with 400 µL of measuring solution. It consisted of 0.42 mM phosphoenolpyruvate and 0.18 mM NADH in a 50 mM HEPES buffer pH 7 containing 13 mM $MnCl_2$, 50 mM KCl and 0.13 % (w/v) BSA. The absorbance at 340 nm was determined using a Beckman Coulter DU 800 UV/VIS spectrophotometer before addition of 0.5 µL of a solution containing the coupling enzymes (PK: 682 U mL^{-1}; LD: 990 U mL^{-1} from rabbit muscle, Sigma-Aldrich). After incubation of the sealed cuvette at 30°C for 45 min the absorbance at 340 nm was measured again and the concentration of UDP was calculated by the difference in absorbance before and after enzyme addition.

One Unit of *Gm*SuSy used in batch or fed-batch conversions for nothofagin production was defined as the amount of enzyme producing 1 µmol NAD^+ per minute under following conditions: 0.2 mM UDP, 300 mM sucrose, 50 mM HEPES, pH 7.5, 13 mM $MgCl_2$, 50 mM KCl, 0.13% BSA and 20% (v/v) DMSO.

Photometric assay for *Gm*SuSy sucrose cleavage activity

The measurement of linear initial rates for sucrose cleavage by *Gm*SuSy were performed by coupling the production of UDP-glucose to NADH formation using human UDP-α-D-glucose 6-dehydrogenase (hUGDH).[4] The oxidation of one molecule UDP-glucose to UDP-α-D-glucuronic acid is accompanied with the reduction of two NAD^+ molecules to NADH (ε = 6220 $M^{-1}cm^{-1}$) which is monitored photometrically at 340 nm. Concentrations of UDP and sucrose as well as the exact buffer conditions are listed at the respective experiment. The reaction volume was typically 1 mL and conversions were performed in 1.5 mL reaction tubes at 30°C in a thermomixer at 400 rpm. The conversion was started by adding *Gm*SuSy. At least 4 samples (typically every 10 min) of 150 µL were withdrawn during the linear initial rate of the reaction. The reactions were stopped by heating the aliquots to 95°C for 5 min. Precipitated protein was removed by 20 min of centrifugation at room temperature and 13200 rpm. 100 µL of the supernatant were mixed with 400 µL of measuring solution containing 2.5 mM NAD^+, 0.05 % Triton™ X-100 and 100 mM HEPES pH 8.0 in a Half Micro Cuvette. The absorbance at 340

nm was determined using a Beckman Coulter DU 800 UV/VIS spectrophotometer before addition of 1.5 mU of hUGDH. After incubation of the sealed cuvette at 30℃ for 45 min the absorbance was measured again and the concentration of UDP-glucose was calculated by the difference in absorbance before and after hUGDH addition.

1.4 Characterization of single enzymes

Determination of pH-profiles

The standard protocol of photometric *Os*CGT activity assay was modified by using a buffer mixture of 12.5 mM HEPES, 12.5 mM Tris and 25 mM CAPS of the respective pH. Reaction buffers were prepared from pH 6.0 to 11.0 in steps of 0.5 pH units. The HEPES concentration of the measuring solution was increased from 50 to 100 mM and it contained 16.25 mM $MgCl_2$ instead of 13 mM $MnCl_2$. The actual pH of the reaction mixture was determined as the average of pH measurements at the beginning and at the end of the observed time span.

Measurements of linear initial rates for sucrose cleavage by *Gm*SuSy were performed at 30℃ by using the standard protocol of the photometric *Gm*SuSy activity assay. The reactions mixtures contained 100 mM sucrose, 2 mM UDP and 20 mM of buffer. 20 mM MES (pH 4.5 - 7.0), 20 mM HEPES (pH 7.0 - 8.5) and 20 mM CHES (pH 8.5 - 10.5) buffers were prepared in steps of 0.5 pH units. The actual pH of the reaction mixture was determined as the average of pH measurements at the beginning and at the end of the observed time span.

Linear initial rates for sucrose synthesis by *Gm*SuSy were measured with the standard photometric assay at 30℃ using 15 mM fructose and 2 mM UDP-glucose in the buffers described for sucrose cleavage. Average pH during conversion was again calculated as average from samples withdrawn at beginning and end of the reaction.

Determination of equilibrium constants (K_{eq})

Equilibrium constant of *Os*CGT was determined by running standard glycosylation reaction (80 mU mL^{-1} *Os*CGT, 5 mM phloretin, 4.75 mM UDP-glucose) until no further conversion was observed. A standard buffer (50 mM HEPES, pH 7.5, 50 mM KCl, 13 mM $MgCl_2$, 0.13% (w/v) BSA, 20% (v/v) DMSO) was used. From all samples phloretin and nothofagin as well as UDP and UDP-glucose concentrations were determined on HPLC using the appropriate protocols. However, quantification of UDP failed and therefore only nothofagin, phloretin and UDP-glucose concentrations could be used for K_{eq} determination.

To test if there is any reverse activity detectable a conversion of 5 mM phloretin and 5 mM UDP-glucose (100 mU mL^{-1} *Os*CGT) was completed as described and subsequently another 5 mM UDP were added to test for deglycosylation of nothofagin. Therefore samples were taken as described over 12 hours and phloretin and nothofagin concentrations were determined per HPLC. Furthermore nothofagin and UDP were used directly as substrate in a reverse reaction using 200 mU mL^{-1} *Os*CGT (1 mM nothofagin, 1 mM UDP, 50 mM HEPES, pH 7.5, 13 mM $MgCl_2$, 50 mM KCl, 0.13% (w/v) BSA, 5% (v/v) ethanol). Nothofagin was extracted from an almost quantitative conversion of phloretin by

*Os*CGT and contained therefore still small amounts of phloretin. Samples were taken over 23 hours and the analyzed by HPLC for phloretin production.
Sucrose cleavage (5 mM sucrose, 5 mM UDP) and synthesis (5 mM fructose and 5 mM UDP-glucose) were catalyzed by 50 mU mL^{-1} *Gm*SuSy using otherwise the same conditions as in *Os*CGT conversions. UDP-glucose concentrations were measured in all samples per HPLC. Comparable to *Os*CGT experiments UDP could not be quantified and concentrations of UDP, sucrose and fructose had to be calculated from starting concentrations and final UDP-glucose level. For K_{eq} calculations data from sucrose cleavage and synthesis was combined.

Determination of half saturation constants

Half saturation and phloretin inhibition constant of *Os*CGT were determined by measurement of linear initial rates at 30℃ in 50 mM HEPES, pH 7.5 containing 50 mM KCl, 13 mM $MgCl_2$, 0.13% (w/v) BSA and 20% (v/v) DMSO using the HPLC-assay. For each substrate 10 different concentrations in a suitable range were used. Phloretin was varied from 0.01 - 10 mM (4 mU mL^{-1} *Os*CGT, 2 mM UDP-glucose) and UDP-glucose was varied from 0.005 - 5 mM (4 mU mL^{-1} *Os*CGT, 1 mM phloretin).
For *Gm*SuSy half saturation constants were determined in sucrose cleavage and synthesis direction. All conversions were made with 2 mU mL^{-1} *Gm*SuSy at 30℃ with 20 mM HEPES, pH 7.5 as buffer. Nine different concentrations of each substrate were used and all activities were measured using the appropriate photometric assays. Sucrose was varied from 5 - 300 mM (2 mM UDP), UDP from 0.0025 - 5 mM (400 mM sucrose), fructose from 0.05 - 30 mM (2 mM UDP-glucose) and UDP-glucose from 0.01 - 5 mM (40 mM fructose).

Influence of cosolvents on *Os*CGT and *Gm*SuSy activity

To test the effect of cosolvents on *Os*CGT and *Gm*SuSy (sucrose cleavage) activity initial rate measurements were done at different concentrations of ethanol or DMSO. Reference measurements for *Gm*SuSy (2 mU mL^{-1}, 0.2 mM UDP, 100 mM sucrose, 50 mM HEPES pH 6.5, 10 mM $MgCl_2$) were made without any organic solvent and those for *Os*CGT (0.2 mU mL^{-1}, 0.6 mM UDP-glucose, 0.1 mM phloretin, 50 mM HEPES pH 7, 13 mM $MnCl_2$, 50 mM KCl, 0.13% (w/v) BSA) were done at 5% of the respective solvent. *Gm*SuSy and *Os*CGT activity were measured using described photometric assays.

Determination of enzyme stability

Enzymes were incubated for certain times (0-24 h) in the final reaction mix at 30℃ and 400 rpm on a thermomixer but without UDP (*Gm*SuSy) or UDP-glucose (*Os*CGT), respectively. Reactions were started by addition of UDP or UDP-glucose and the loss of activity in dependence of incubation time was used to fit first order inactivation kinetics for calculation of half-lifes. In case of *Os*CGT glycosylation activity was measured by HPLC (15 mU mL^{-1}, 5 mM phloretin, 1 mM UDP-glucose) and sucrose cleavage of *Gm*SuSy was determined photometrically (2 mU mL^{-1}, 100 mM sucrose, 1 mM UDP). The buffer for *Gm*SuSy was 50 mM HEPES, pH 7.5 with 13 mM $MgCl_2$, and 20 % (v/v) DMSO and that for *Os*CGT additionally contained 50 mM KCl and 0.13 % (w/v) BSA.

1.5 *Os*CGT conversions coupled with *Gm*SuSy

Batch conversions of *Os*CGT coupled with *Gm*SuSy

Standard reaction mixtures contained 5 mM phloretin, 100 mM sucrose, 0.5 mM UDP, 13 mM $MgCl_2$, 50 mM KCl, 0.13 % (w/v) BSA, 20 % (v/v) DMSO and 50 mM HEPES buffer pH 7.5. Enzymatic conversions were initiated by the addition of the respective amount of *Os*CGT and *Gm*SuSy. Reactions were performed in 1.5 mL reaction tubes at 30℃ in a thermomixer at 400 rpm. Samples were analyzed with the HPLC-based *Os*CGT assay to determine the concentrations of phloretin and nothofagin.

For optimizing the conversion conditions the concentrations of the substrate were varied separately: UDP (0.005 - 1 mM; 10 mU mL^{-1} *Os*CGT, 10 mU mL^{-1} *Gm*SuSy), sucrose (5 - 500 mM; 10 mU mL^{-1} *Os*CGT, 10 mU mL^{-1} *Gm*SuSy) and phloretin (5 - 30 mM; 190 mU mL^{-1} *Os*CGT, 120 mU mL^{-1} *Gm*SuSy). On variation of sucrose and UDP only initial nothofagin production rates were analyzed during the first two hours (3-4 samples) but on variation of phloretin complete conversion curves over 24 hours were recorded.

For comparison of phloretin conversion by *Os*CGT with and without coupling to UDP-glucose recycling with *Gm*SuSy instead of 0.5 mM UDP 6 mM UDP-glucose were used. Both enzymes were applied at 50 mU mL^{-1}.

Fed-batch conversions of *Os*CGT coupled with *Gm*SuSy

Phloretin concentrations of the batch conversion were modified for the fed-batch experiments. The reaction solution contained 300 mM sucrose, 1 mM UDP, 13 mM $MgCl_2$, 50 mM KCl, 0.13 % (w/v) BSA, 20 % (v/v) DMSO in 50 mM HEPES buffer (pH 7.5). Separate reactions with 5 and 10 mM phloretin, respectively were made in a total volume of 2 mL. Reactions were started by enzyme addition (100 mU mL^{-1} *Os*CGT/*Gm*SuSy) and incubated at 30℃ and 400 rpm on a thermomixer. Directly after taking samples phloretin and nothofagin concentrations were measured on HPLC and upon depletion of phloretin, new substrate was added from a stock of 500 mM phloretin in DMSO. The increase of substrate concentration was thereby equivalent to the initial concentrations of 5 and 10 mM, respectively. Separate reactions were made with and without feeding of fresh enzyme (50 mU mL^{-1}) at the same time as the substrate feed.

1.6 Product isolation and identification by HPLC and NMR

The product of phloretin conversions by *Os*CGT has previously been identified as nothofagin using ^1H and ^{13}C NMR.[5] Therefore 1 mM phloretin was converted to nothofagin (2 mM UDP-glucose in 50 mM HEPES pH 7, 13 mM $MnCl_2$, 50 mM KCl, 0.13% BSA and 5% (v/v) ethanol) and the resulting product was almost quantitatively extracted from the reaction solution by repeated extraction with ethyl acetate. After removing the solvent under reduced pressure the product was dissolved in $MeOD_4$ for NMR analysis. HPLC retention time of the product of coupled conversion of *Os*CGT with *Gm*SuSy was compared with nothofagin for product verification.

Nothofagin was purified by preparative reversed phase C-18 HPLC. Proteins were removed from phloretin conversions by centrifugal concentrators with a Molecular Weight Cut Off of 10 kDa. The HPLC system from Knauer Technologies (smartline pump 1000 V7603, smartline DAD UV detector

2600, manual 6 port injection valve) was equipped with a C18-reversed phase VP 125/21 Nucleodur 100-5 C18ec column. Water containing 0.1% TFA was used as solvent A and methanol as solvent B. Separation of nothofagin from other compounds was achieved by a 20 min long gradient from 20 to 100% B followed by 6 min of isocratic flow at 100% B. Separation was monitored by UV-detection at 288 nm. Fractions containing nothofagin were pooled, methanol was evaporated under reduced pressure and water was removed by freeze drying.

2 Results

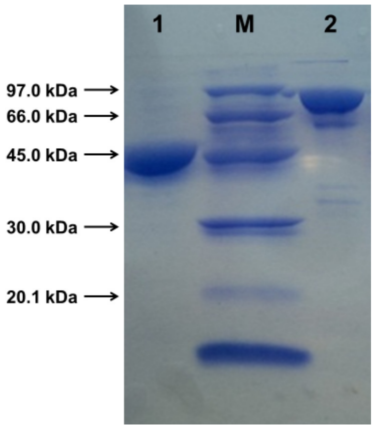

Figure S1: SDS-PAGE of enzymes purified by *Strep-tag* affinity chromatography: Lane 1: *Os*CGT (51.3 kDa); Lane 2: *Gm*SuSy (94.1 kDa); Lane M: Low Molecular Weight standard (GE Healthcare)

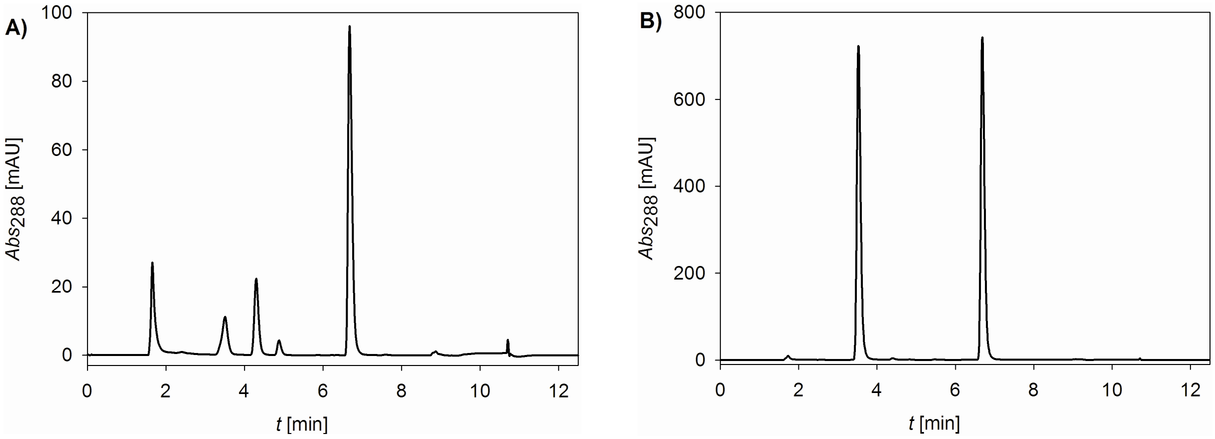

Figure S2: Separation of phloretin from its glycosides using RP-HPLC: A) mixture containing non-binding compounds like UDP(-glucose) (1.7 min), 3'-*C*-glycoside nothofagin (3.5 min), 2'-*O*-glycoside phlorizin (4.3 min), 4'-*O*-glycoside (4.9 min) and phloretin (6.7 min); B) partial phloretin conversion using *Os*CGT and *Gm*SuSy contains only 3'-*C*-glycoside nothofagin (3.5 min), phloretin (6.7 min) and small amounts of UDP(-glucose)

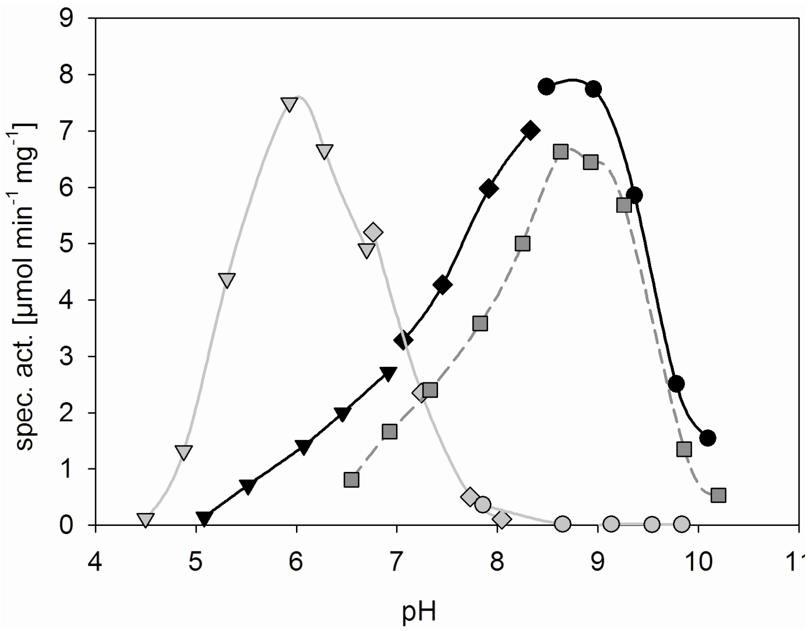

Figure S3. pH profiles for nothofagin synthesis by *Os*CGT (dark grey, dashed line), sucrose cleavage (light grey) and sucrose synthesis (black) by *Gm*SuSy using a mixture of HEPES, Tris and CAPS (square), MES (triangle), HEPES (diamond) or CHES (circle) as buffer.

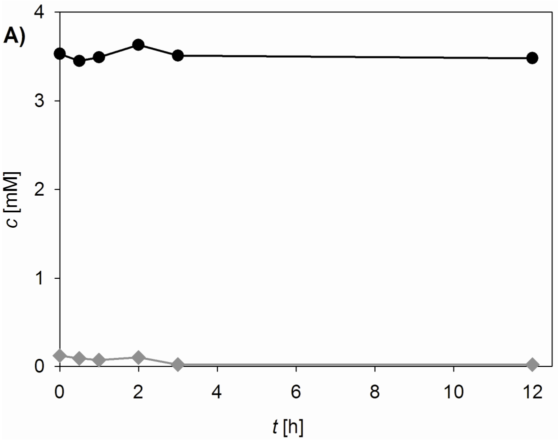

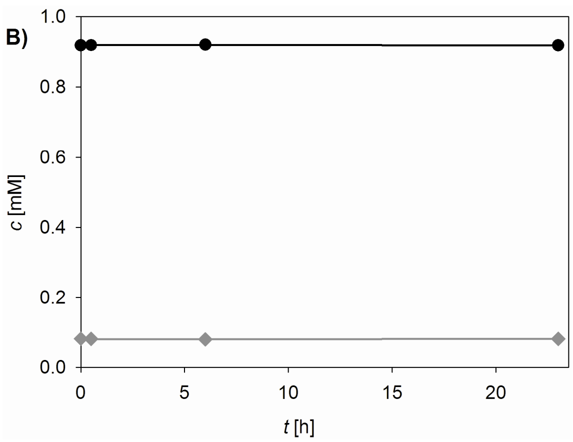

Figure S4: Deglycosylation of nothofagin (black) to phloretin (grey) by *Os*CGT could not be observed: A) 5 mM phloretin and 5 mM UDP-glucose were almost completely converted by 100 mU mL^{-1} *Os*CGT (not shown). When adding further 5 mM UDP (dilution) no deglycosylation of nothofagin was observed; B) Incubation of ~1 mM nothofagin containing some phloretin with 1 mM UDP (200 mU mL^{-1} *Os*CGT) did also not result in any further phloretin production.

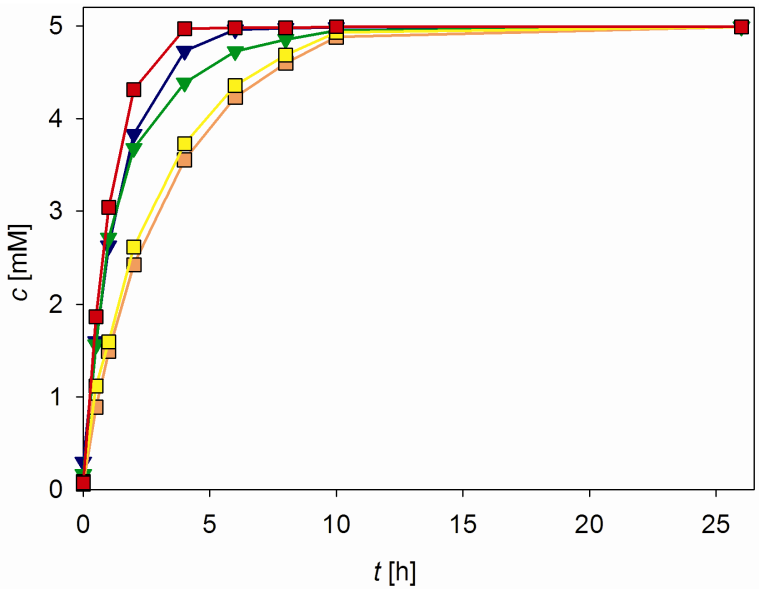

Figure S5. Nothofagin production in *Os*CGT catalyzed conversions of 5 mM phloretin (100 mM sucrose) at various conditions: 50 mU mL^{-1} *Os*CGT, 6 mM UDP-glucose (green); 50 mU mL^{-1} *Os*CGT/*Gm*SuSy, 6 mM UDP-glucose (blue); 50 mU mL^{-1} *Os*CGT/*Gm*SuSy, 0.5 mM UDP (orange); 50 mU mL^{-1} *Os*CGT, 250 mU mL^{-1} *Gm*SuSy, 0.5 mM UDP (yellow); 250 mU mL^{-1} *Os*CGT, 50 mU mL^{-1} *Gm*SuSy, 0.5 mM UDP (red)

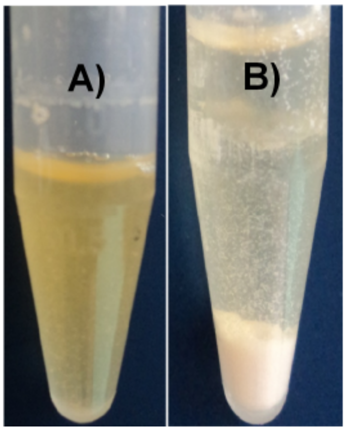

Figure S6: Increased solubility of nothofagin compared to phloretin: A) In a fed-batch conversion of *Os*CGT and *Gm*SuSy with feeding of 5 mM phloretin and enzyme in total 45 mM phloretin were applied and fully converted to nothofagin causing only enzyme to precipitate; B) In an identical solution without enzyme most of the applied 45 mM phloretin was precipitated.

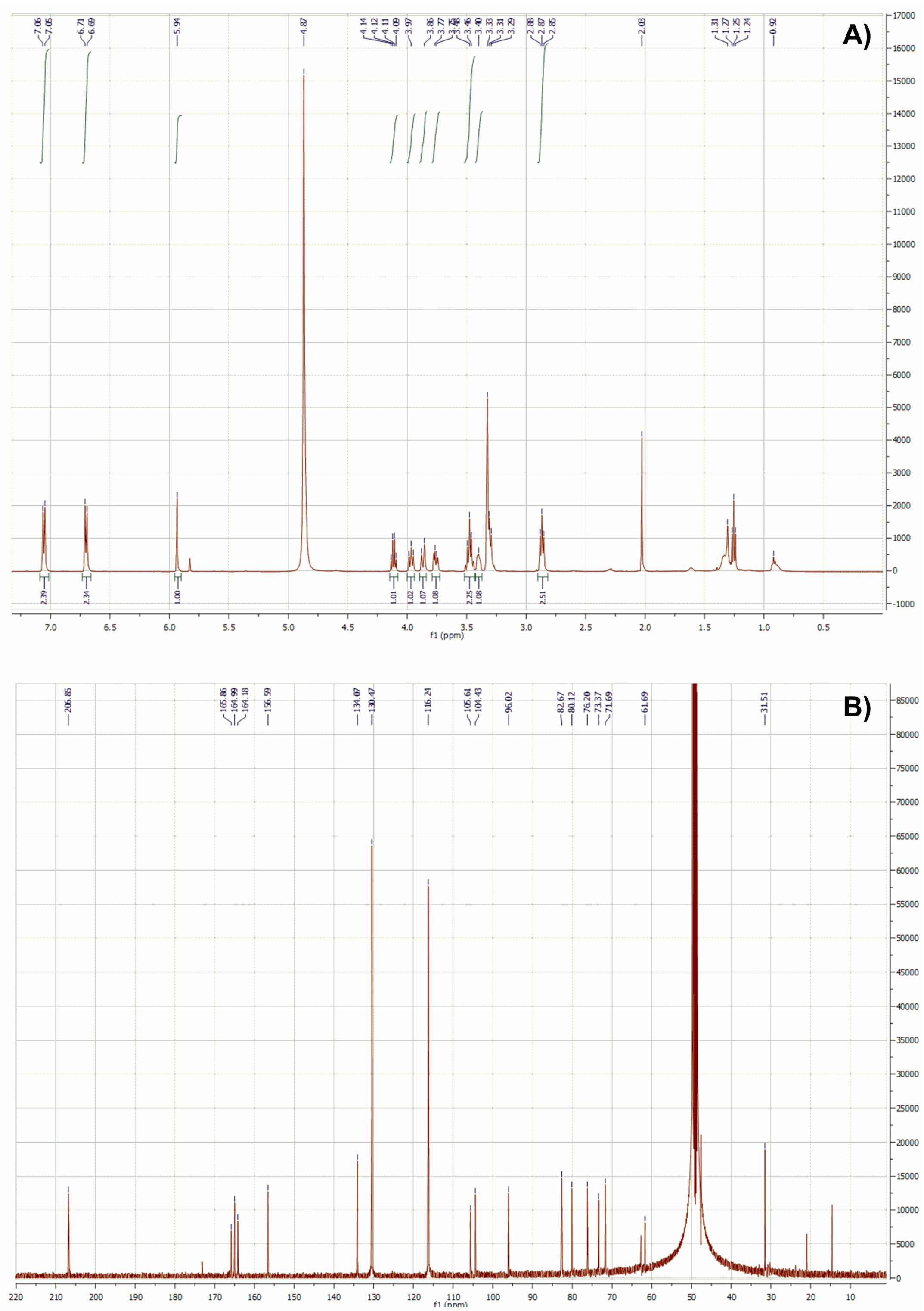

Figure S7: ^{1}H A) and ^{13}C-NMR B) of nothofagin after extraction with ethyl acetate from enzymatic conversion of phloretin by *Os*CGT.

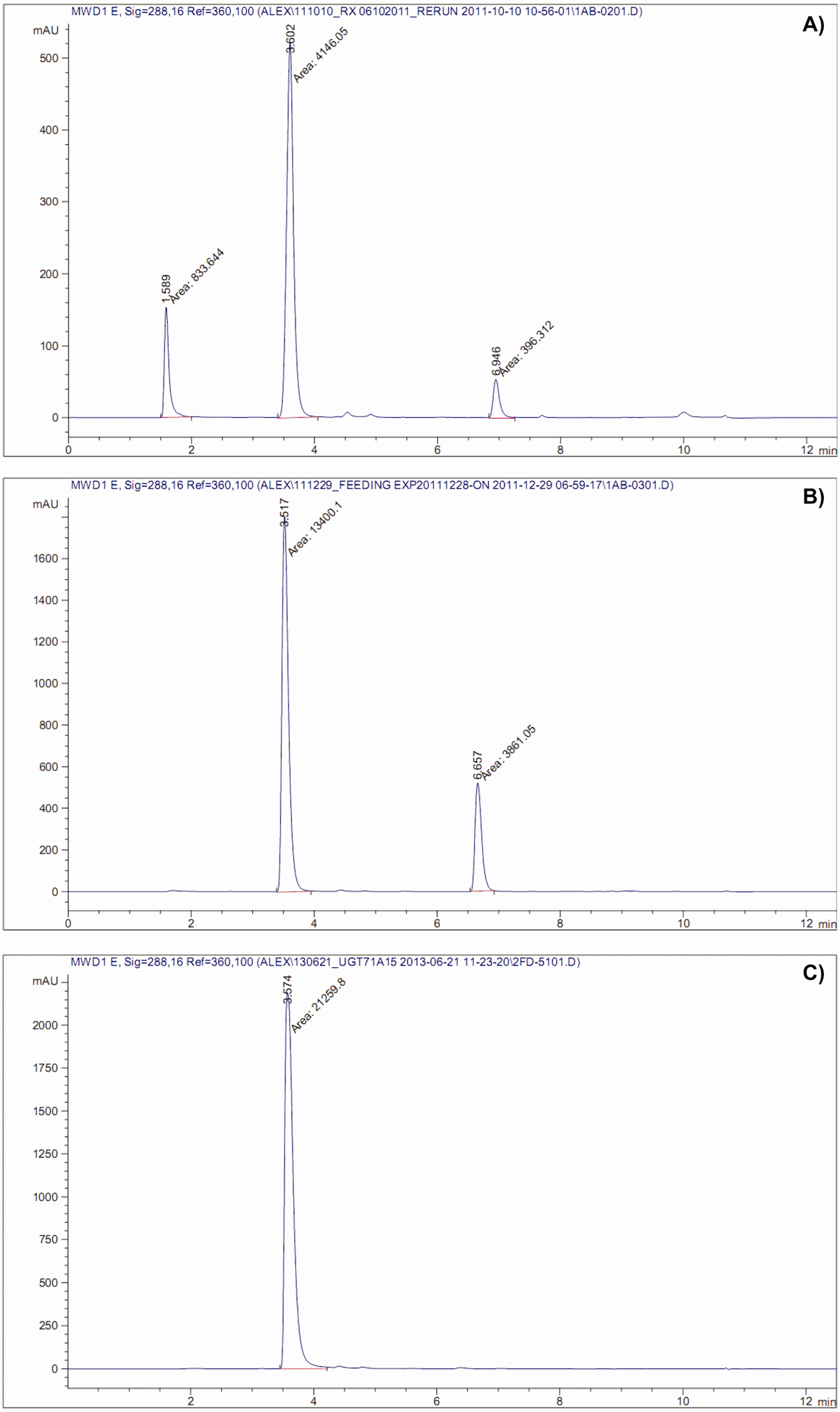

Figure S8: HPLC chromatograms (UV detection at 288 nm) showing nothofagin peak at ~3.5 min either from authentic standard A), after partial phloretin conversion in a coupled reaction of *Os*CGT and *Gm*SuSy B) and after purification by reversed phase HPLC C).

3 References

[1] M. Brazier-Hicks, K. M. Evans, M. C. Gershater, H. Puschmann, P. G. Steel, R. Edwards, *J. Biol. Chem.* **2009**, *284*, 17926-17934.

[2] D. P. Dixon, T. Hawkins, P. J. Hussey, R. Edwards, *J. Exp. Bot.* **2009**, *60*, 1207-1218.

[3] S. Gosselin, M. Alhussaini, M. B. Streiff, K. Takabayashi, M. M. Palcic, *Anal. Biochem.* **1994**, *220*, 92-97.

[4] S. Egger, A. Chaikuad, K. L. Kavanagh, U. Oppermann, B. Nidetzky, *Biochem. Soc. Trans.* **2010**, *38*, 1378-1385.

[5] A. Gutmann, B. Nidetzky, *Angew. Chem.* **2012**, 124, 13051–13056; *Angew. Chem. Int. Ed.* **2012**, *51*, 12879-12883.
